# Results after implementation of a protocol on the incidence of urinary tract infection in an intensive care unit**^1^**

**DOI:** 10.1590/1518-8345.0866.2804

**Published:** 2016-09-09

**Authors:** Anna Letícia Miranda, Ana Lúcia Lyrio de Oliveira, Daiana Terra Nacer, Cynthia Adalgisa Mesojedovas Aguiar

**Affiliations:** 2 MSc, RN, Hospital das Clínicas, Universidade Federal de Minas Gerais, Belo Horizonte, MG, Brazil.; 3 PhD, Professor, Universidade Federal de Mato Grosso do Sul, Campo Grande, MS, Brazil.; 4 MSc. RN, Hospital Universitário Maria Aparecida Pedrossian, Campo Grande, MS, Brazil.; 5 Master's student, Universidade Federal do Mato Grosso do Sul, Campo Grande, MS, Brazil. RN, Hospital Sociedade Beneficente de Campo Grande, Santa Casa, Campo Grande, MS, Brazil.

**Keywords:** Reprodutive Tract Infections, Practice Guideline, Critical Care

## Abstract

**Objective:**

to compare the results of urinary tract infection incidence, by means of the rate of indwelling urethral catheter use, and to identify microorganisms in urine cultures and surveillance cultures before and after the implementation of a clinical protocol for intensive care unit patients .

**Method:**

urinary tract infection is defined as a positive urine culture > 105 CFU/mL, notified by the hospital infection control service, six months before and after the implementation of the protocol. The sample consisted of 47 patients, 28 reported before and 19 after implementation. The protocol established in the institution is based on the Ministry of Health manual to prevent healthcare-related infections; the goal is patient safety and improving the quality of health services.

**Results:**

a negative linear correlation was observed between the later months of implementation and the reduction of reported cases of urinary tract infection, using the Spearman rank order coefficient (p = 0.045), and a reduction in the number of urine culture microorganisms (p = 0.026) using the Fisher exact test.

**Conclusion:**

educational interventions with implementation protocols in health institutions favor the standardization of maintenance of the invasive devices, which may reduce colonization and subsequent infections.

## Introduction

Patient safety has been greatly emphasized by health institutions, with targets set by global organizations which must be reached in order to minimize the risk of healthcare-related harm^1^. 

Healthcare-associated infections (HAIs) are considered infections which occur after the admission of the patient to the hospital. Of the HAIs, urinary tract infections (UTI) are one of the most prevalent, and have the greatest potential for prevention, due to their relationship with urethral catheterization^2^.

Although cases of patients with urinary infection present a lower mortality rate (0.28%), a notification of an infection rate of 25% to 60%^3^.

A UTI is identified in high numbers among risk groups, such as: pregnant women, the elderly, diabetics, and patients with coronary artery disease. The periodic recurrence of infection, and its inadequate treatment, leads to an increase in contagion that could favor epidemics. Thus, recent studies validate the notification of UTI cases with 10^3^CFU counts in critically ill patients, with or without symptoms^3^.

In order to minimize the error occurring during patient care, there must be a care management process in hospitals, with continuing education processes and implementation of protocols, or clinical guideline recommendations, identifying actions to prevent harm derived from patient care^4^.

The American organization, the Institute for Healthcare Improvement (IHI), in 2001, implemented a package of preventive measures based on evidence, called a "bundle", with the aim of reducing deaths from harm and infections during care^5^.

This process was recently validated in Brazil and demonstrated good results in the reduction of HAIs case reporting.

## Method

This was a pre-experimental study, type 0X0, performed in a high complexity philanthropic hospital in the state of Mato Grosso do Sul, Brazil, with most of the care by the Unified Health System in the intensive care unit for adult patients, consisting of 15 beds. Data collection was performed by means of analysis of the electronic medical record (EMR) for access to demographic, epidemiological and clinical data of patients, associated with the secondary data from the Hospital Infection Control Service (HICS). The results of urinary tract infection incidence density from May 2013 to May 2014 was compared, referring to the six months before and after the implementation of the compliance protocol; November was deleted, as this was the month of implementation, due to the process of adaptation.

The protocol consists of four measures that are the main Ministry of Health recommendations for preventing urinary tract infections related to care. It must be ensured that each patient receives: 1- an aseptic technique during catheter insertion. 2- Daily review of the need to maintain the catheter, removal of it as soon as possible. 3- Avoidance of unnecessary use of long dwelling urinary catheters. 4- Maintain the use of urinary catheters (UC) only when based on recommended guidance documents^2^.

From the decision of the need for the patient to undergo an invasive procedure, the professional must perform the steps correctly described the checklist issued by the institution, based on the Ministry of Health guideline, such as: organization of all required materials, hand washing, correct periurethral hygiene, identification and validation of the procedure in the nursing process manual.

The urethral catheter compliance protocol was implemented within the routine daily care, being completed daily by HICS professionals who identify nonconforming items related to the maintenance of the device. The following items were evaluated: 1. The correct fixation of the catheter. 2. The device identification (brand name, date of insertion of the catheter, lumen size) 3- Maintenance of urine collector bag below the level of the bladder. 4- Urine volume below 2/3 to prevent reflux. 5- Clear urinary flow. 6 Proper disinfection plug for sample collection (urine culture and NUA) 7- Daily justification to maintain the catheter through nursing prescription (nursing care systematization),with the prescription recorded in the electronic medical record.

The patients in the sample defined with urinary tract infection by HICS demonstrated positive urine culture laboratory tests > 105 CFU / ml, associated with signs and symptoms, based on the manual of diagnostic criteria of healthcare-related infections ^1^. All patients in the study used the indwelling catheters, included as one of the criteria for asymptomatic UTI.

According to secondary data, the patients presented with: fever > 38°C, presence of leukocyte esterase or nitrite in urine analyses (NUA), and the presence of pyuria in urine, with new collection of urine culture and device exchange occurring as routine.

After notification of urinary tract infection, to obtain the incidence density (ID) of UTI, of Catheter-associated Urinary Tract Infection, requires the following formula by HICS professionals, based on the Ministry of Health.



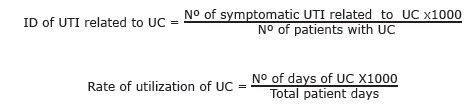



The study population consisted of 47 patients with UTI notification by HICS, 28 patients notified before and 19 patients after the implementation of the protocol.

The study excluded patients with urinary tract infection under 18, indigenous, and those who died after at least 72 hours in the ICU.

The evaluation was performed by means of a Student's t-test and Spearman linear correlation test, and the results of other variables were analyzed by a Chi-square test (Fisher's exact) statistical, with 5% significance level shown by figures and tables.

This research followed Resolution No. 466 of December 12, 2012, the Plenary of the National Health Council, and was approved by the Research Ethics Committee of the Federal University of Mato Grosso do Sul under protocol No. 790073, September 12, 2014, after authorization by the institution.

Because this study required the collection of secondary data and access to electronic medical records, without direct contact with the patient, the free informed consent form was dismissed.

## Results

Table 1 shows the conditions of the risk of ICU patients: 34% developed acute renal failure requiring dialysis therapy, 19% required parenteral nutrition, and 77% were taking corticosteroids. Of the total of 47 patients, 83% were not responsive to volume hypotension, requiring a continuous infusion of vasoplegic amines, and 45% were diabetic and needed glycemic correction.

**Table 1:** Characteristics of patients admitted to the ICU with urinary tract infection, before and after the implementation of the protocol, according to study variables, Campo Grande MS, Brazil, 2013 -2014

Variables			Time in relation to implementing the clinical protocol				Valor de *p*		
			Before		After		Total		
		Incident density of urinary tract infection	13,85±2,07		9,88±2,54				0,254*
			n	%	n	%	n	%	
Characteristics									
	Gender								
		Female	15	53,6	10	52,6	25	53	0,592^†^
		Male	13	46,4	9	47,4	22	47	
	Age group								
		< 60 years	12	42,9	10	52,6	22	47	0,562^†^
		> 60 years	16	57,1	9	47,4	25	53	
Risk Conditions									
	Hemodialysis catheter								
		Yes	7	25	9	47,4	16	34	0,102^†^
		No	21	75	10	52,6	31	66	
	Vasoactive medications								
		Yes	23	82,1	16	84,2	39	83	0,589^†^
		No	5	17,9	3	15,8	8	17	
	Corticosteroids								
		Yes	19	67,9	17	89,5	36	77	0,083^†^
		No	9	32,1	2	10,5	11	23	
	Parenteral nutrition								
		Yes	6	21,4	3	15,8	9	19	0,465^†^
		No	22	78,6	16	84,2	38	81	
	Diabetes Mellitus								
		Yes	12	42,9	9	47,9	21	45	0,497^†^
		No	16	57,1	10	52,6	26	55	
Laboratory Exams									
	Blood culture								
		Positive	15	53,6	15	78,9	30	64	0,122^†^
		Negative	13	46,4	4	21,1	17	36	
	Outcome								
	Death								
		Yes	15	53,6	11	57,9	26	5	0,503^†^
		No	13	46,4	8	42,1	21	45	

The results are in relative frequency (absolute frequency) or mean ± standard deviation. * p-value in Student t- test. † p-value in Fisher's chi-square or exact test.

The age of the patients in this study ranged from 18 to 92 years, there was no statistical difference regarding their gender. All 47 patients with UTI diagnosis used prophylaxis for gastric ulcer (100%) and underwent urethral catheterization (100%) mainly during their ICU admission, and the catheter was removed or changed after positive urine culture, or after macroscopic presence of pyuria or sediment in urine displayed on the closed system by professionals during the multi-disciplinary discussions.

It was observed that the total sample, 30 patients (64%), had a positive blood culture and 26 (55.3%) eventually died.

Regarding the results of UTI incidence density before and after the implementation of the protocol, there was a reduction of cases from 13.85 ± 2.07 to 9.88 ± 2.54, but not significant when analyzed using the student t-test (p = 0.254).

**Figure 1:**
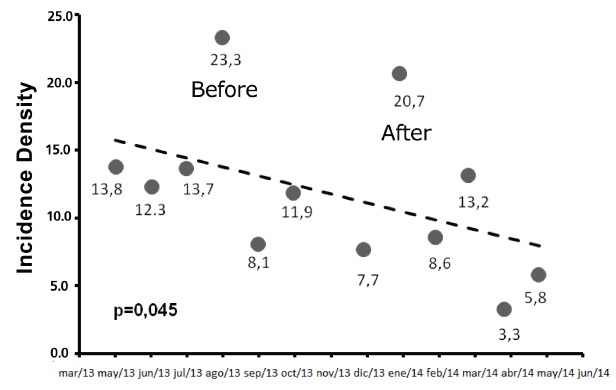
Scatterplot showing the linear correlation between the months of study and urinary tract infection incidence density, among patients from ICU, Campo Grande MS, Brazil, 2013 -2014

The reduction in UTI incidence density was not statistically significant according to the Student's t-test when compared to Spearman's test. A significant but moderate negative linear correlation was found between this variable and the subsequent months evaluated in the study (p = 0.045, r = -0.580), leading to a reduction of reporting of cases of UTI by the Hospital Infection Control Service. The result is shown in Figure 1.

**Figure 2:**
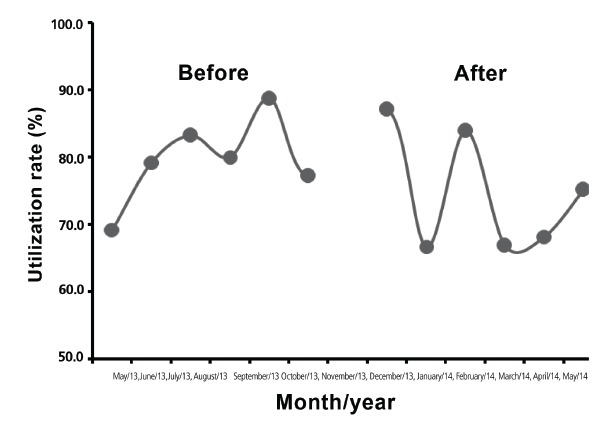
Graph showing the urethral catheter utilization rate in each of the months in this study, before and after the implementation of the protocol, among ICU patients, Campo Grande MS, Brazil, 2013 -2014

Figure 2 demonstrates that there was no statistical difference between the two groups with urethral catheters, referring to the time before and after the implementation of the protocol (p = 0.303), 79.58 ± 2.65%, 74.66 ± 3 67% due to the severity of ICU patients and the need to maintain the device to aid in therapy.

The number of microorganisms isolated in the urine culture of patients admitted to the ICU, before and after the implementation of the protocol, are demonstrated in Table 2.

**Table 2:** Results of urine culture microorganisms in patients admitted to the ICU before and after implementation of the protocol, Campo Grande MS, Brazil, 2013 - 2014

Variables							
		Before (n=28)		After (n=19)		Total (n=47)	
		n	%	n	%	n	%
Microorganisms isolated in the urine culture (p=0.026)							
	Only 1	13	46.4	15	78.9	28	59.6
	> 1	15	53.6	4	21.1	19	40.4
Infectious bacteria							
	Acinetobacter sp	2	7.1	0	0.0	2	4.3
	Candida albicans	8	28.6	5	26.3	13	27.7
	*Candida* não-albicans^*^	5	17.9	0	0.0	5	10.6
	Candida glabrata	0	0.0	2	10.5	2	4.3
	Candida krusei	0	0.0	2	10.5	2	4.3
	Candida tropicalis	1	3.6	3	15.8	4	8.5
	*Enterococcus/VRE* ^†^	07/1	28.6	4	21.1	12	25.5
	Enterobacter sp	1	3.6	0	0.0	1	2.1
	Escherichia coli	4	14.3	3	15.8	7	14.9
	*Klebsiella pneumoniae* (p=0.122)	13	46..4	4	21..1	17	36..2
	Pseudomonas aeruginosa	2	7.1	1	5.3	3	6.4
	Proteus sp	1	3.6	0	0.0	1	2.1
	Trichosporon spp	2	7.1	0	0.0	2	4.3

The results are in relative frequency (absolute frequency), p-value of the Fisher exact test, or chi-square test.

* *Candida albicans* is not related to non-identification of the species prior to the implementation of the protocol at the institution.

† VRE: vancomycin-resistant *enterococcus*.

There was a significant association between the reduction in the number of microorganisms found in urine cultures from 53.6% to 21.1% (Fisher's exact test, p = 0.026)and the time in relation to the implementation of the protocol,.

The reduction of the multirrestient bacterium, *Klebsiella pneumoniae,* after the implementation of the protocol was of great impact for the study of reduction (46.4% to 21.1%) (Fisher's exact test, p = 0.122), p <0.05.

Regarding surveillance cultures collected by means of an anal swab of patients on admission to the intensive care unit, Table 3 demonstrates the effectiveness of surveillance caution with *Kblebsiella pneumoniae* identification with patients; there was no statistical significance between colonization and evolution to positive urine culture for this agent.

**Table 3:** Evaluation of association of the anal swab and urine culture positive for the micro-organism, *Klebsiella pneumoniae*, in patients admitted to the ICU, Campo Grande, MS, Brazil, 2013-2014

Variable		Anal Swab for *Klebsiella* pneumoniae			P Value	
		Positive (n=10)		Negative (n=37)		
		nº	%	nº	%	
Urine culture for *Klebsiella* pneumoniae						0.460
	Positive	5	50.0	12	32.4	
	Negative	5	50.0	25	67.6	

## Discussion

The educational intervention administered by nurses in clinical practice by means of training of the institution's health professionals, a has demonstrated favorable results, as noted in the reduction in reported cases of incidence density of UTI by the Hospital Infection Control Service.

Although the first six months after the implementation of the protocol were not statistically significant, according to the student's t-test (p = 0.254), the best results achieved occurred in April of 2014, with an incidence density of 3.3, and 5.8 in May of 2014, analyzed by Spearman's test (p = 0.045), which corroborates the results recommended by ANVISA, reaching the incidence rate of 3.1 -7.4 / 1000 catheter / day^2^.

In recent scientific studies on the incidence of UTI density, predictive values and the results of assistance in the world are quite variable. In Canada, the prevalence rate ranges from 4.7% to 7% among pregnant women with UTI. In Ethiopia, the incidence density of up to 23.9% was reported; in the southern part of Nigeria a prevalence of UTI of 86.6% was noted. This shows that the patient's risk factors, associated with a lack of human resources and technology, have a strong influence on the surge in infections, and consequently prolonged periods of hospitalization and death^6^.

A study performed in Iran, by six university hospitals, on rates of infection and bacterial resistance associated with invasive devices showed a incidence density rate of 8.99 per 1000 catheter days^7^.

Regarding studies on the effectiveness of protocols, adherence to an instrument in a health institution can take up to two years to deliver results, and educational interventions to transform variable results to permanent ones, and that the reduction, if it is not statistically significant, is beneficial in reducing the length of stay of patients being treated for infections, and consequently mortality^8^.

This study regarding the results of the implementation of a protocol suffered from some interference, such as the small sample size, which justifies further research over a longer period of time, to evaluate the subsequent months; although statistics in health institutions about patient safety and adverse events related to care are very recent.

An increased incidence density was observed in a few months, which may be associated with seasonal factors such as sector refurbishments. After adherence to the protocol at the institution, new staff were hired, which improved the quality of surveillance and underreported cases, restructuring of the program, and the HICS database. It is known that there is a weakness among health professionals regarding real charting and the absence of data recorded from fragile sources. There are various weaknesses for a researcher in clinical practice, which can explain this increase as a weak aspect of the study.

Many studies emphasize that patients with advanced age, diabetes, and those who are female (because of their anatomy) are groups with a higher predisposition to urinary tract infection. However, in our study, when comparing the groups there was no significant association observed between gender and age (p ranging from .465-.083); this demonstrates that regardless of patient risk factors, severity in the intensive care unit promotes health care-related infections^3^.

Regarding the utilization rate of the urethral catheter, there were no statistical differences between the time before and after the protocol implementation (p=0,303), which highlight the severity of ICU patients and the need for maintaining the device in order to support the therapy.

In a retrospective study on the risk factors associated with urinary tract infection (UTI) based on a *Clinical Practice Guideline*, intermittent catheterization was strongly recommended as the first choice in patients with a need for catheterization; however for those patients who needed more than three intermittent catheterizations/day or patients with paraplegia who are at high risk of urinary tract dysfunction, the urethral catheterization became vital^9^.

Therefore, considering that in the intensive care unit there was no difference in reducing the utilization rate of urethral catheters associated with incidence density of UTI in the moments before and after the protocol implementation, the study allows the possibility of implementing the protocol on the wards due to the lower severity of these patients in these units; the need to maintain the urethral catheter must be contested daily by the nurse, exploring alternative methods such as intermittent catheterization and frequent diaper changes.

The purchase of scales in the institution in which the study was conducted allowed the removal of invasive devices of patients in the ward and in the intensive care unit, in those lower severity patients, while maintaining the quality of strict hydric control.

The implementation of this protocol at the institution was able to reduce the number of microorganisms in the urine culture (Fisher's exact test, p = 0.026), reflecting the reduction in hospital costs for treating patients with septic shock from a urinary infection.

Reducing the number of microorganisms after the implementation of the protocol is extremely important, because the reduction of colonization prevents infection. In a multicenter study performed in 2012, it was observed that cases of funguria were resolved without treatment in 76% of patients in a large clinical cohort study. The treatment of choice for funguria was two weeks on an antifungal, which was similar to the placebo group, so the early removal of the catheter was the most promising intervention^10^.

The surveillance cultures collected at admission to the intensive care unit demonstrated that the patient's isolation criteria transferred from other hospitals, with prolonged hospitalization, with over 72 hours of stay in the emergency room or a positive swab culture being effective.

A recent study published in the *Journal of Infectious Disease* claimed that gram-negative bacillus (GNB) and producing bacteria carbapenemases (KPC) such as *E. Coli* e *Klebsiella pneumoniae,* found in rectal swabs of previously colonized patients, presented a significantly increased risk of urinary infection by the same microorganism^11^.

In this study, it was observed that there was no statistical significance between patients with a positive swab for *Klebsiella pneumoniae* in the urine culture, which highlight that the proper maintenance of the invasive medical devices and isolation of patients are essential factors for the reduction of cases of cross-transmission caused by healthcare professionals, and not only by the patient's clinical condition^12^.

In another study, it was clearly stated that the urinary tract is a very frequent source of infection and that more than 20% of the patients will not be affected by urinary tract infection if the screening and isolation by the results of the rectal swabs are completely reliable^11^.

Despite not having performed molecular tests in this study to identify the concomitant microorganisms in urine and blood cultures, the total sample of patients with urinary tract infection (64%) had a positive blood culture; of which (14.7%) had the same microorganism in the urine.

Recent studies state that isolates of *Candida spp* blood were identical to those found in urine culture, confirmed by molecular biology and that patient with candiduria have a high risk for candidemia^13^. The percentage of deaths in this study was (55.3%); using a statistically significant sample size, patients who were initially reported with urinary tract infection, later evolved into serious complications and reporting of bloodstream infection, followed by death.

## Conclusion

It was concluded in this study that the implementation of the protocol ("bundle") in intensive care units presents a negative linear correlation to the reduction of cases of incidence of urinary tract infection in the course of months.

The sample was statistically significant in order to reduce the number of microorganisms found in the urine culture.

Continuing education and protocol maintenance may present favorable effects on the reduction of reported cases of UTI.
